# Orthotopic kidney transplantation survival and complications: systematic review and meta-analysis

**DOI:** 10.1080/2090598X.2022.2090133

**Published:** 2022-06-17

**Authors:** Carlos Alfredo Castillo-Delgado, Herney Andrés García-Perdomo, Mireia Musquera, Antonio Alcaraz

**Affiliations:** a Division of Transplant Surgery, Department of General Surgery, Hospital General Plaza de la Salud, Santo Domingo, Dominican Republic; b Division of Urology/Urooncology, Department of Surgery, UROGIV Research Group, Universidad del Valle, Cali, Colombia; c Division of Kidney Transplant Surgery, Department of Urology, Hospital Clinic – University of Barcelona, Barcelona, Spain

**Keywords:** Orthotopic kidney transplantation, systematic review, meta-analysis, kidney graft, overall survival

## Abstract

**Purpose:**

To determine graft and patient survival and adverse events in patients who undergo orthotopic kidney transplantation.

**Methods:**

We performed a systematic review and meta-analysis. We search in Medline, Embase, and Central from inception to nowadays. We included observational studies with patients who undergo orthotopic kidney transplantation. The primary outcomes were overall patient and graft survival. We pooled the information in a frequency meta-analysis with a 95% CI. We analyzed bias with the STROBE statement.

**Results:**

Of the 106 papers initially retrieved, four met the inclusion criteria. Vascular and urinary tract complications were reported in 19% and 15%, respectively. The overall patient survival was 92% 95%CI (88% to 95%), I2 = 0%, and the overall graft survival was 88% 95 CI (83% to 91%), I2 = 0%.

**Conclusion:**

Our analysis showed a high survival rate in patients and kidney grafts after orthotopic kidney transplantation, with a similar complication rate compared to a heterotopic kidney transplant.

## Introduction

Currently, End-Stage Renal Disease (ESRD) is a worldwide highly prevalent condition, especially for those patients with comorbid conditions such as diabetes, obesity, and hypertension [1,2]. In these patients, the endothelium damage with subsequent atherosclerosis and vascular stenosis makes it not suitable for heterotopic kidney transplantation [3].

Kidney transplants had played an important and relevant role in the past few decades, since 1954 when Joseph Murray performed the first successful [4]. In addition, with the constant evolution of immunosuppressive therapy, and the improvement of the surgical technique, the list of contraindications has decreased. Consequently, we have a high percentage of patients ongoing for the third and fourth kidney transplants [5].

Sometimes, heterotopic kidney transplantation is not possible because of retained bilateral iliac fossa for a previous kidney transplant, urinary diversion, or vascular alterations. Accordingly, an orthotopic kidney transplant (OKT) might be an excellent alternative [6].

The orthotopic kidney transplant was first described by Gil-Vernet et al. in 1989 and consisted of implanting the kidney graft through a retroperitoneal approach, mainly using the left renal vein, the left renal pelvis-ureter, and the splenic artery [7,8]. This Technique requires more surgical skills in comparison with the heterotopic kidney transplant; hence, currently, only a few centers manage this operation.

Nevertheless, controversy exists about managing a patient with a contraindication for using the iliac fossa for the implantation because there are only a few publications about orthotopic kidney transplants. Accordingly, the long-term results are not well known, and the technique requires a highly skilled surgeon.

In the literature there are few series of OKT, the largest has 216 cases. Therefore, this study aimed to determine the adverse events and graft and patient survival in patients who undergo orthotopic kidney transplantation.

## Methods

We performed this review according to the recommendations of the Cochrane Collaboration [9] and following the PRISMA Statement [10].

### Eligibility criteria

Study designs: We included observational studies.

Participants: Studies including patients who undergo orthotopic kidney transplantation.

Primary outcome: Overall survival and graft survival

Secondary outcome: Adverse effects

Timing: For all outcomes, studies should have at least a one-month follow-up.

Exclusion criteria: We excluded case series with less than four patients and a single case report. No language restrictions were applied.

### Information sources

We searched MEDLINE (OVID), EMBASE, LILACS, and the Cochrane Central Register of Controlled Trials (CENTRAL) from inception to nowadays (Appendix 1). To ensure literature saturation, we will scan references from relevant articles identified through the search, conferences, thesis databases, Open Grey, Google Scholar, and clinicaltrials.gov. We will contact authors by e-mail in case of missing information. There will be no setting or language restrictions.

### Data collection

Two researchers reviewed each reference by title and abstract. Then they scanned full texts of relevant studies, applied pre-specified inclusion criteria, and extracted the data. Disagreements were resolved by consensus, and when the disagreement could not be solved, a third reviewer dissolved the conflict.

Using a standardized form, two trained reviewers independently extracted the following information from each article: study design, geographic location, authors’ names, objectives, inclusion and exclusion criteria, sample size, losses to follow up, timing, outcomes, and association measures.

### Risk of bias assessment

We used the STROBE statement to assess the risk of bias.

### Data analysis/Synthesis of results

The statistical analysis was executed in R. We performed a meta-analysis of proportions with the command metaprop and the method inverse (logit transformed proportions). Information was pooled with a random effect meta-analysis according to the heterogeneity expected. A priori, We considered a high clinical heterogeneity and a wide proportion of variation among studies. In addition, the results were reported in forest plots of the estimated effects of the included studies with a 95% confidence interval (CI). Finally, heterogeneity was evaluated using the I^2^ test. For the interpretation, we determined that the values of <50% and >50% in the I^2^ test corresponded to low and high levels of heterogeneity, respectively.

### Sensitivity analysis

We performed sensitivity analysis, extracting weighted studies and running the estimated effect to find differences.

### Subgroup analysis

None

## Results

### Study selection

**Figure 1.**
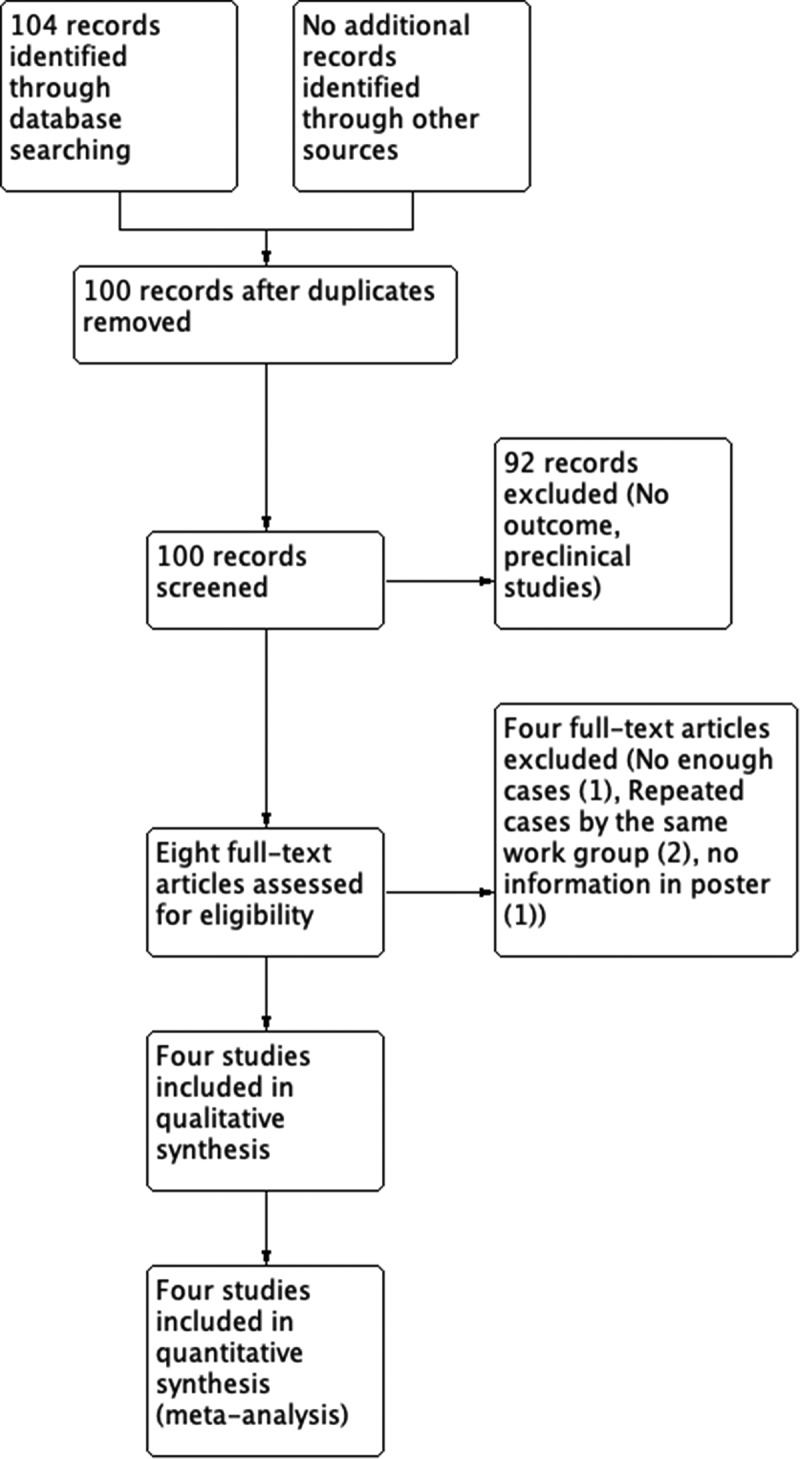
Flowchart of included studies.

We found 106 studies with search strategies described previously. After duplicate exclusion and full-text evaluation, four studies met inclusion criteria [2,6,11,12] in qualitative and quantitative analyses. One center had two publications [7] (Figure 1).

### Characteristics of included studies

We included four studies, which comprised 243 patients. Three of them were from Spain, one from the United States of America. Male was the predominant patient’s gender, with a mean age of 47.5 years (Range 4.2 to 73.6). The terminology used for ESRD etiology was not the same in these studies. The orthotopic kidney transplant indications were primarily severe iliac atherosclerosis; nonetheless, Musquera et al. presented a comparison between two periods, where during the first period 84.8% of the cases were performed in an elective indication [6,7]. The graft proceeded in most cases from deceased donors (Table 1).

**Table 1.** Characteristics of included studies.

Study	n	Age	Gender	Indication of OKT	Type of Donor
Musquera, 2010 A	139	36 yr (11–67)	No Data	Elective surgery (100%)	Living Donor (63%); Deceased Donor (37%)
Musquera, 2010 B	84	46.7 yr (4.2–73.6).	68 M /16 F.	Severe iliac atherosclerosis (41.7%), bilaterally retained iliac fossae from a previous kidney transplant (28.9%), elective indications for OKT (14.5%)	Living Donor (7.1%) Deceased Donor (92.9%)
Hevia 2014	9	48.5 yr (23.6–63.4)	M(78%) F (22%)	Unsuitable Iliac Region (67%) (occupation by previous transplants, severe atheromatosis, thrombosis), LUT abnormalities/ Urinary Diversion (33%)	Deceased Donors (100%)
De Gracia 2007	6	50.1 yr (41–62)	M (4) F (2)	Severe iliac atherosclerosis (83.3%), bilaterally retained iliac fossae from a previous kidney transplant (16.6%)	Deceased Donors (100%)
Paduch 2001	5	56 yr (47–69)	M (4) F (1)	Severe iliac atherosclerosis (40%), bilaterally retained iliac fossae from a previous kidney transplant (40%%), aortoiliac occlusion (20%)	Deceased Donors (60%) living donor (40%)

^a^OKT: Orthotopic kidney transplant, LUT: Lower urinary tract.

### Risk of bias

There was an unclear risk of bias for the objective, participants, and study design since there was no information regarding these items. Two studies (De Gracia et al. and Paduch et al.) reported the patient’s information; however, there was not an appropriate description of the statistical analysis [11,12]. The rest of the items were classified as low risk of bias since they were appropriately described (Table 2).

**Table 2.** Risk of bias assessment. The Strengthening the Reporting of Observational Studies in Epidemiology (STROBE) table. Green: fully answered, Yellow: moderately, and Red: not described.

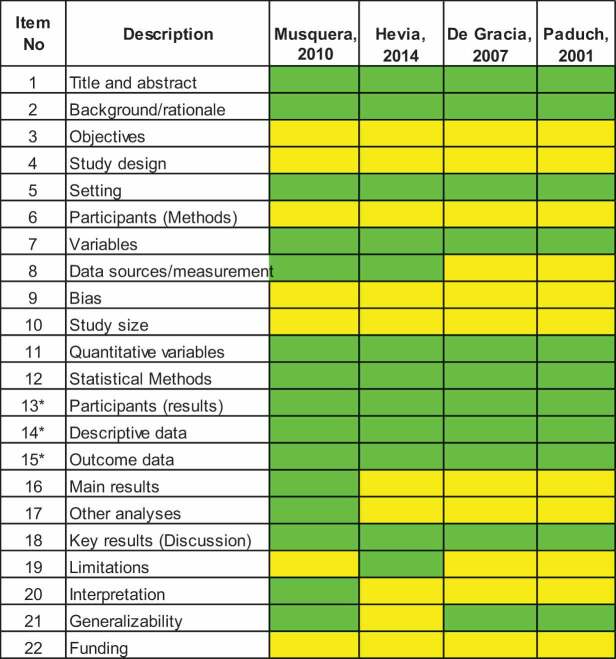	

### Outcomes

#### Follow up

Postoperative follow-up varied according to the author, Hevia, et al. presented a mean follow-up of 91.8 months, Paduch, et al. from six months to five years, De Gracia et al., 24 months, while Musquera et al., with the longest follow-up of 20 years.

### Overall patient and graft survival

**Figure 2.**
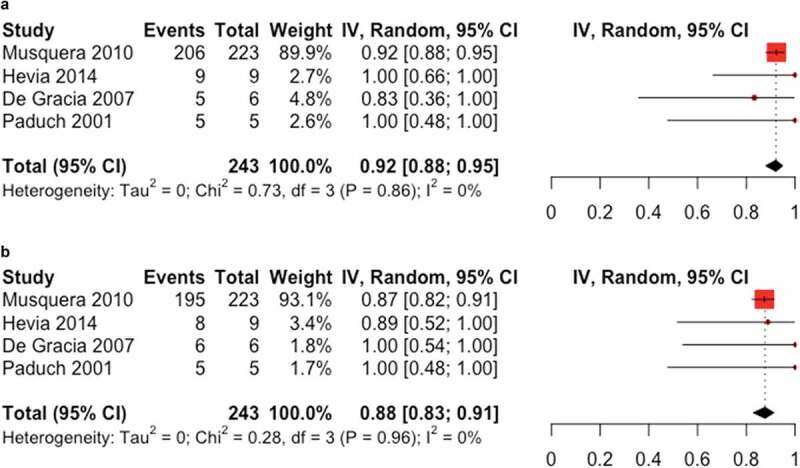
(**a**) Forest plot of overall survival. (**b**) Forest plot of graft survival.

The four included papers described overall patient and graft survival. We found a pooled frequency of 92% 95% CI (88% to 95%); I2 = 0% for patient survival (Figure 2a) and a pooled frequency of 88% 95% CI (83% to 91%); I2 = 0% graft survival(Figure 2b).

### Secondary outcomes

We found 19% and 15% of vascular and urinary complications, respectively. Kidney transplant outcomes are described in Table 3.

**Table 3.** Secondary outcomes forest plots.

Outcome	Number of studies	Frequency	95%CI	I2
Overall Survival	4	92	88 to 95	0%
Overall Kidney Graft Survival	4	88	83 to 91	0%
Delayed Graft Function	3	20	08 to 43	0%
Trasnplantectomy	3	6	03 to 10	0%
**Vascular Complications**				
Vascular Complications	4	19	07 to 44	80%
Arterial Stenosis	4	10	02 to 37	79%
Arterial Trombosis	4	6	02 to 15	36%
**Urinary Complications**				
Urinary Complications	4	15	11 to 20	0%
Urinary Fistula	4	7	05 to 11	0%
Urinary Obstruction	4	5	01 to 15	42%

### Sensitivity analysis

Although the more weighted study was Musquera et al. 2010, results did not significatively changed when performing the sensitivity analysis.

## Discussion

Orthotopic kidney transplantation has been reserved for those patients with contraindication for heterotopic one, due to atheromatosis, vascular abnormalities, or even urinary tract problems, becoming an excellent and safe alternative to heterotopic transplantation. The current systematic review and meta-analysis show a high survival rate in patients who underwent orthotopic kidney transplantation with a 92% overall patient survival, with an overall graft survival rate of 88%. This rate is considerably high, especially in a setting of patients with increased mortality risk due to multiple comorbidities [2].

Delayed graft function (DGF) was found in 20% of cases, similar results obtained by other authors, such as Seth et al. who conducted a retrospective study of 95 transplant patients, where they found 21% DGF [13,14]. Risk factors for DGF are several, related to the donor (donation after brain and cardiac death, cold ischemic time, shipping distance, donor age, BMI, race, right donor nephrectomy and open nephrectomy), to the recipient (time before transplantation in dialysis, previous kidney transplants, panel reactive antibody, ABO incompatibility, history of diabetes, BMI, recipient sex, and race) and to perioperative risk factors (induction medications and types of anesthetics) [15]. The lack of data in the studies about primary graft dysfunction did not allow us to draw a strong conclusion.

On the other hand, the rate of vascular complications in heterotopic kidney transplants is around 3–15% according to some published series [13,14,16], while in our patients it achieves slightly higher results with 19%, with arterial stenosis and vascular thrombosis being most complications with 10% and 6%, respectively. Others vascular complications corresponded to arterial bleeding due to technical failure and venous thrombosis.

The most frequent cause of vascular stenosis in the literature is due to technical failure, generally located at the level of the anastomosis. Other associated causes are vascular injury during preservation or due to the use of clamps, and torsion, kinking or angulation of the artery and possibly atherosclerosis [17]. Being the probable causes of the rate of arterial stenosis in our study, since, as we have commented, they are patients with important vascular deterioration. Some authors presented the management, from angioplasty, open surgery to transplantectomy.

In addition, we found a similar rate of urinary complications in both groups, being 15% in orthotopic kidney transplantation and 1–15% in heterotopic transplantation, according to some published series. Urinary fistula and obstruction are the two most frequent categories [18,19]. Ureteral obstruction/stenosis is associated with technical failure or surgical complications, such as hematomas, lymphoceles in the early postoperative period, while in late stenosis the etiology remains more uncertain, associated with factors such as DGF, ureteral duplication, donor age, number of the arteries and the presence of urinomas [20]. Although the incidence of ureteral complications has decreased recently due to improvements in extraction techniques, knowledge and preservation of the ureteral vasculature, and the proper use of double-J catheters, this is especially important when a kidney is removed with surgical purposes, especially for orthotopic transplant, where the proximal portion of the native ureter is used, which has its proximal irrigation affected, and is also where the anastomosis is performed.

Although case reports were not included, we also wanted to describe a few characteristics from them. Three manuscripts reported cases from young people with specific conditions indicating the orthotopic transplant, such as multiple pelvic arteriovenous malformations, congenital abnormalities, multiple pelvic and abdominal surgeries, and twin pregnancy [18,21,22]. In addition, two papers described people older than 60 years with multiple comorbidities and severe aortoiliac atherosclerosis [23,24]. Rodrigues et al. described four cases (two young people and two older than 60 years old) with severe atherosclerosis [25]. Furthermore, Chan et al. showed three patients (two young and one older than 60 years old) with inferior vena cava (IVC) thrombosis or stenosis [26]. On the other side, Novotny et al. described a patient with a papillary renal cell carcinoma recurrence who underwent radical nephrectomy, along with an OKT [27]. Unfortunately, all those case reports did not show any overall or kidney survival information and associated complications.

## Recommendations

In the first place, all patients who undergo a renal transplantation must have their vascular anatomy evaluated by Angio CT scan, to confirm the possibility of performing a heterotopic transplant. Furthermore, if it is contraindicated, to assess the feasibility of an orthotopic transplant, before excluding from the transplant list.

Secondly, since orthotopic renal transplantation is an option for patients with a contraindication for heterotopic transplantation, because of atheromatosis, vascular abnormalities, or even urinary tract problems, it is technically complex, so it is recommended with a previously detailed technique study, and, accordingly, perform them in high-volume centers.

## Strengths and limitations

Our main limitations were that we only found four studies that met the eligibility criteria, mostly Spanish papers. Furthermore, the two longest series reported are from the same center.

Secondly, there was a high clinical heterogeneity due to the complexity and specificity of the procedure, requiring a certain level of expertise. Subsequently, these results should not be extrapolated to every team implementing this procedure.

## Conclusion

Orthotopic kidney transplantation is a feasible, safe, and reproducible alternative in patients with contraindication for heterotopic transplantation, with low rate of complications, and a high rate of recipient and graft survival. Nonetheless, we need more studies to accomplish these critical outcomes.

## Appendix Search StrategyMedline (Ovid)

((orthotopic kidney transplant*).mp or (orthotopic renal transplant*).mp or (orthotopic kidney graft transplant*).mp or (orthotopic renal graft transplant*).mp) AND (survival.mp or (overall survival).mp or complication*.mp or (adverse effect*).mp or (adverse event*).mp).

## Central (Ovid)

((orthotopic kidney transplant*).mp or (orthotopic renal transplant*).mp or (orthotopic kidney graft transplant*).mp or (orthotopic renal graft transplant*).mp) AND (survival.mp or (overall survival).mp or complication*.mp or (adverse effect*).mp or (adverse event*).mp).

## Embase

(‘orthotopic kidney transplant*’:ti,ab or ‘orthotopic renal transplant*’:ti,ab or ‘orthotopic kidney graft transplant*’:ti,ab or ‘orthotopic renal graft transplant*’:ti,ab) AND (survival:ti,ab or ‘overall survival’:ti,ab or complication*:ti,ab or ‘adverse effect*’:ti,ab or ‘adverse event*’:ti,ab).
